# Patient voices and student insights into LGBTQ+ healthcare: a call for equitable healthcare through medical education

**DOI:** 10.1080/10872981.2024.2405484

**Published:** 2024-09-17

**Authors:** Michael X Fu, Simisola Onanuga, Xinyu Ye, Raksha Aiyappan, Tangming Zou, Susan Smith, Ana Baptista

**Affiliations:** a Medical Education Research Unit, Faculty of Medicine, Imperial College London, London, UK; b Nuffield Department of Medicine, University of Oxford, Oxford, UK; c Lee Kong Chian School of Medicine, Nanyang Technological University, Singapore, Singapore; d Tan Tok Seng Hospital, Singapore, Singapore

**Keywords:** Medical, education, students, LGBTQ+, equity

## Abstract

**Purpose:**

Lesbian, gay, bisexual, transgender, queer, and other sexual and gender diverse (LGBTQ+) individuals have health needs specific to their identities. However, they face discrimination and cis-heteronormativity in most patient-provider interactions, which often translate into poor healthcare. Evidence suggests doctors are inadequately trained to care for LGBTQ+ patients. Medical students are well-placed as the future workforce to establish affirming behaviours. This study garners LGBTQ+ patients’ healthcare experiences, where limited qualitative evidence exists, and explores whether students have insight into these experiences.

**Method:**

Thirty LGBTQ+ patients and twenty students, evenly divided between Singapore and the United Kingdom (UK), two legally and culturally different countries, consented to semi-structured interviews in 2022 to evaluate their LGBTQ+ healthcare perceptions. Thematic analysis was conducted using a collaborative, iterative process involving five investigators, with frequent auditing of data interpretation.

**Results:**

Most patients described implicit biases with a lack of support and professionalism from doctors, hindering health outcomes. Patients experienced misgendering and a lack of recognition of sexual and gender diversity; students appreciated the need to acknowledge patient identity. Although perceptions surrounding certain themes were similar between patients and students in both countries, patients’ voices on the complexity and dissatisfaction of gender-diverse care contrasted with students’ lack of insight on these themes. Singapore patients were more concerned with sociolegal acceptance affecting health needs, whilst UK patients noted more nuanced barriers to healthcare. Although many students were unsure about specific health needs and perceived a lack of training, they expressed willingness to create an equitable healthcare environment.

**Conclusions:**

LGBTQ+ patients provided powerful narratives on discrimination surrounding their healthcare needs. To address these, medical students must be encouraged by healthcare educators to develop identity-affirming behaviours as future change-makers and challenge cis-heteronormative views. Alongside vital institutional changes tailored to each country, patients’ and students’ collective action would create meaningful educational opportunities to reach culture change.

## Introduction

I caught a nasty respiratory infection and ended up in my local ICU [intensive care unit] for a week. Obviously, I didn’t know what was going on [sedated in ICU]. The issue was there was a lot of deference in the hospital to my mum and dad, which was very nice but to the exclusion of my husband, who legally is my next of kin … For example, [when] visiting in intensive care, who was going to be around for the consultant-led ward round and who was going to be fed back to and given an update; or, if there was a meeting to be held in the family room, which was never a place for happy news: there was always the deference to my parents, whether that was because they were a generation above or a lack of appreciation for the relationship … Thankfully, they all agreed on the right plan. But actually, at a time when he [my husband] was very vulnerable and feeling very scared, instead of looking at him as the next of kin, he was seen as complementary or downgraded, I would say.

No, it was not some fleeting relationship. We were a married couple: this is the person you should actually lead your interactions with. I don’t think there was an intention to exclude him, but I think it was an overlooking of the meaningfulness of us as a family. I don’t think it was a conscious thing - I think it was an unconscious bias toward the significance of our relationship. It was the fact that my husband was listed as my next of kin, and I don’t think that that was honoured. I think that was because he was another man. I don’t think they would have done that to a married heterosexual couple, and we were a married, gay couple - I draw this as a comparison.

This was in a central London teaching hospital seven years ago, so we’re not going back two decades and into the middle of the countryside; we were actually in a centre of excellence that teaches the next generation of healthcare professionals. You model what you see. If that is what people see as they are training as professionals, then that’s actually the behaviours that you might include into your own practice, consciously or not.Vignette 1: patient UK15 describes the need to talk about promoting equitable healthcare

Lesbian, gay, bisexual, transgender, queer, and other sexual and gender diverse (LGBTQ+) individuals experience significant health inequalities [1–3]. LGBTQ+ individuals also face discrimination from individual and systematic stigmatisation [4,5]. Differences in the societal and political landscapes worldwide may tailor the healthcare received by LGBTQ+ individuals. For example, in Singapore, societal attitudes towards LGBTQ+ individuals remain largely conservative in contrast to nations such as the UK, where sociolegal acceptance is more progressive [6]. Although Singapore legalised same-sex sexual activities in 2022, same-sex marriages remained unrecognised [7], with a lack of rights to adoption and fertility treatment compared to heterosexual partners [8]. This contrasts with the UK, where parenting options for LGBTQ+ couples, including donor insemination and adoption, are openly available to explore [9]. Further, only 33% of Singaporeans supported the legalisation of same-sex marriage compared to 66% of adults in the UK in 2024 [10]. The tendency of Singaporeans to hold homonegative attitudes through disapproval or rejection of LGBTQ+ individuals’ identities [11] has potential repercussions. Ethnographic interviews showed that gay Singaporean men were hesitant to ‘come out’ as gay due to fear of prosecution and discrimination [12].

The health inequalities that LGBTQ+ individuals experience can be exacerbated by minority stressors, discrimination and inequity in treatment [13,14]. However, the Singaporean Medical Council’s and the United Kingdom (UK) General Medical Council’s guidelines detail expectations that patients should be treated equally, without discrimination or bias based on sexual orientation or gender identity [15,16]. Country-level recommendations similarly reflect a call to action on improving health provisions and its inclusivity of LGBTQ+ patients, although from separate entities: a governmental call to action for the UK [17], compared to peer-led and community-based organisations in Singapore [18].

Yet evidence suggests doctors are inadequately equipped to care for LGBTQ+ patients, from reasons such as training deficits regarding LGBTQ+ care [19], discrimination, or discouraging healthcare-seeking behaviours [20]. Even for doctors who do not actively discriminate, implicit biases may introduce unintentional inequality in treatment and interaction with LGBTQ+ patients [21,22] and further worsen health outcomes [23].

The foundations of doctors’ clinical communications and practice are formed within their education. Exposure to culture change and bias reductions can be introduced whilst in training [23]. As the future workforce, medical students have the potential to develop identity-affirming behaviours and address stigma and implicit biases early in practice [23]. However, a lack of training has been suggested to contribute to barriers to doctors providing sufficient care [24]. This qualitative study aimed to generate nuanced perspectives of the health needs and past experiences of LGBTQ+ individuals in Singapore and the UK, two contrasting countries regarding the sociocultural landscape for LGBTQ+ individuals. From the qualitative data, this study also explores how medical education could be adapted for future doctors to provide them with greater insights into LGBTQ+ patient experiences.

## Materials and method

### Design

Ethics approval was obtained from the Imperial College Research Ethics Committee (21IC7342) and the Nanyang Technological University Institutional Review Board (IRB-2021-521). The patient interview guide (Supplementary Material 1) was developed through discussions between co-investigators following a literature search on LGBTQ+ health needs. Semi-structured interviews began by exploring the patients’ general experiences of being LGBTQ+ in society, followed by questions about their health needs and experiences. Guiding questions supported this, and further probing questions were dependent on responses. Discussions with LGBTQ+ non-profit organisations- and medical education experts ensured the validity of the questions.

The student interview (Supplemental Material 2) was developed through co-investigators reflecting upon themes from patient interviews. Students were asked to provide their perceptions of these health needs (unprompted response), and if not offered, they were asked about specific health needs (prompted response). Students’ perceptions of how well these needs were met were probed. Further questions were asked about students’ education about LGBTQ+ health needs at medical school. All questions were intended to be open-ended and avoided leading language [25]. If interviewees provided vague responses, further questions were asked, provided they were comfortable offering clarification. Interviews were conducted in English since the UK and Singapore are primarily English-speaking countries, and interviewees were expected to be fluent in this language. To better understand local jargon, two investigators conducted each interview in the investigators’ own country.

### Procedures

The patient interview was piloted with one LGBTQ+ identifying person from each country to check for understanding and accuracy, and their suggestions were incorporated into the final version. Between October 2021 and January 2022, volunteer patients were consecutively recruited for 30–60-minute interviews via national LGBTQ+ organisations’ social media channels until fifteen interviews had been conducted in each country. Volunteer students were consecutively recruited for 15–30-minute interviews via social media groups between August and September 2022 until ten students from each medical school had been interviewed. Thematic analyses of the 30 patient and 20 student interviews found that thematic saturation was reached as no new top-level themes emerged from the data for both cohorts [26]. Thus, no further participants were interviewed.

Information sheets were emailed before each interview, and informed consent was obtained to transcribe and securely store transcripts and publish anonymised comments. All interviews were conducted remotely to reach a geographically diverse population under COVID-19 restrictions. Patients and students were offered 15GBP/30SGD and 5GBP/10SGD vouchers respectively. Demographics were collected at the time of the interviews. Member checking was completed, and no participant withdrew or redacted information from their transcript.

### Participants

Eligibility criteria for patients included those over the legal age for providing informed consent: 18 (UK) and 21 (Singapore) years. Patients also needed to identify as LGBTQ+ with experience(s) of LGBTQ±related healthcare in their country of residence. Inclusion criteria for students were 18 years of age and medical student registration at one of the participating medical schools.

### Data analysis

Interviews were transcribed verbatim and anonymised before analysis. Thematic analysis was performed using NVivo software (release 1.6.2, OSR International), and a constructivist paradigm approach was followed [27]. Student investigators collaboratively performed an iterative process of identifying themes and sub-themes, which first represented individual data but became more conceptual with repetitive reviews and coding [28]. This enabled intercoder clarification of themes and codes, strengthening validity and reliability. Discrete ideas from each quotation were only coded once. Discussions between the five investigators identified the most suitable code if an idea fitted more than one code, meaning saturation of the codes from both interview cohorts gave richness and complexity to the data [26]. Staff authors audited the analyses, providing further research triangulation. Interview numbers for each quote were further anonymised before data comparison to minimise potential investigator bias introduced when extracting quotes from specific transcripts. One investigator summarised the sub-themes for each patient and student interview, and then a second investigator validated this by adding or challenging perspectives. A third investigator then cross-referenced critical messages from the patient interviews with themes in the student interviews, highlighting significant inter-country differences, whilst the fourth audited this comparison. References to ‘gender-diverse’ individuals in this manuscript encompass individuals who identify as trans, transgender, and gender-expansive.

### Statement of reflexivity

Throughout the study, conscious reflections on the investigators’ paradigmatic stance were made. Firstly, collaborating with an LGBTQ+ non-governmental organisation to review the interview questions ensured that unconscious assumptions were negated and that additional perspectives from those who worked directly with health-seeking LGBTQ+ individuals were included in more open-ended questions. Further, investigators were enthusiastic towards the subject. The investigators had regular interactions with close friends who identified as LGBTQ+ and experienced a lack of medical education surrounding LGBTQ+ healthcare. The investigators also knew of poor healthcare provisions and discrimination towards LGBTQ+ patients. Therefore, analyses may have been prone to investigators’ assumptions and personal views. Collaborative reflexive exercises were undertaken to minimise this: thematic analysis sessions were conducted with at least two co-investigators present, and regular group discussions under a foundation of trust and culture of mutual responsibility for rigorous and ethical analyses allowed for assumptions to become evident from others’ points of view and ensured that consensus interpretations derived from the data provided a diversity of perspectives [29].

Since investigators were five medical students at different stages of training at the time of interviews, the medical students interviewed may have known or been known to certain investigators. As such, it was ensured that no students were interviewed by an investigator who knew them personally to minimise personal conflicts of interest with the student interviewees that may have affected their disclosures. A potential power dynamic existed among (often younger) medical student interviewers and different LGBTQ+ patient stakeholders; this resulted in an empowering dynamic for patients who perceived it as an important opportunity to have their voices heard systematically, reinforced by the fact that many interviews lasted longer than the expected 60-minutes. Lastly, the pseudo-anonymisation of interviewees using numerical identifiers and further identifier anonymisation during the data analysis stage, where only shorter quotes were available to review compared to entire transcripts, ensured that potential investigator bias towards certain interviewees was minimised throughout data analyses.

## Results

### Demographics

UK patients primarily identified with lesbian, gay or bisexual identities, whereas in Singapore, there was a more diverse demographic of trans and queer individuals (Table 1). Most students did not identify with any LGBTQ+ identity, and students’ year groups varied. Thematic saturation from the codes suggested that, when taken together, the recruited participants (ten students and fifteen patients in each country) provided sufficient data across all themes despite disparities in participant identities, where many participants offered shared experiences from their close contacts who identified as gender-diverse. All interviewees were fluent in English.

**Table 1.** Demographic characteristics of the patients and students interviewed.

Demographic	UK patients, n = 15	SG patients, n = 15	All patients, n = 30	UK students, n = 10	SG students, n = 10	All students, n = 20
*Primary LGBTQ+ identity*						
Lesbian	4 (27%)	0	4 (13%)	0	0	0
Gay	5 (33%)	2 (13%)	7 (23%)	1 (10%)	0	1 (5%)
Bisexual	5 (33%)	5 (33%)	10 (33%)	3 (30%)	0	3 (15%)
Trans	0	4 (27%)	4 (13%)	0	0	0
Queer	1 (7%)	4 (27%)	5 (17%)	0	0	0
Pansexual	0	0	0	1 (10%)	0	1 (5%)
Non-LGBTQ+	0	0	0	5 (50%)	8 (80%)	13 (65%)
Unsure or undisclosed	0	0	0	0	2 (20%)	2 (10%)
*Gender identity*						
Male	5 (33%)	3 (20%)	8 (27%)	3 (30%)	7 (70%)	10 (50%)
Female	9 (60%)	7 (47%)	16 (53%)	6 (60%)	3 (30%)	9 (45%)
Trans male	1 (7%)	0	1 (3%)	0	0	0
Trans female	0	3 (20%)	3 (10%)	0	0	0
Non-binary	0	1 (7%)	1 (3%)	1 (10%)	0	1 (5%)
Gender-fluid	0	1 (7%)	1 (3%)	0	0	0
Age*						
21-30	8 (53%)	9 (60%)	17 (57%)	–	–	–
31-40	3 (20%)	4 (27%)	7 (23%)	–	–	–
41-50	4 (27%)	2 (13%)	6 (20%)	–	–	–
Year of Study*						
2	–	–	–	1 (10%)	5 (50%)	6 (30%)
3	–	–	–	3 (30%)	2 (20%)	5 (25%)
4	–	–	–	1 (10%)	#	1 (5%)
5	–	–	–	0	2 (20%)	2 (10%)
6	–	–	–	5 (50%)	1 (10%)	6 (30%)

*The year of study was asked for students, whilst age was asked for the patients.

#Year 4 in the UK medical school is an intercalated year where students study for another degree, but this does not exist in the Singapore medical school. There are six years in the UK medical school, compared to 5 in the Singapore medical school. To align the clinical studies, Year 4 is omitted from the Singapore medical school and is instead represented as Year 5, and Year 5 in the Singapore medical school is represented as Year 6.

We organised the data from the patient interviews into six themes: acknowledging patient identity, health affected by psychosocial factors, sexual and reproductive health, mental health, gender-diverse health, and support and professionalism. We then compared the patients’ information about each theme to the students’ perspectives. The following findings are consensual themes and sub-themes from the data that apply to both countries except where the country name is explicitly stated.

### Acknowledging patient identity

For LGBTQ+ patients, gender identity and sexual orientation have varying degrees of relevance. At the minimum, appropriate use of correct pronouns and names and respect for patients’ sexual orientation (through affirming language to the patient and practice that affirms their partner) play an essential role in their healthcare experiences. Conversely, many students did not cite acknowledging patient identity until prompted.

Once students mentioned patient identity as being important, the views of students and patients were better aligned. Students in both countries noted that asking for patient pronouns was part of acknowledging patient identity and discussed the importance of regularly asking and establishing correct gender pronouns and how patients can receive that well (Q1, Table 2).

**Table 2.** Representative quotes for each sub-theme of the ‘acknowledging patient identity’ theme cross-referenced to the corresponding text.

Subtheme	Representative quotes	In-text reference
Importance of asking for pronouns	Student UK8: *‘In my experience, like when I was on psychiatry [placement], the psychiatrist will always use people’s preferred pronouns.’*	Q1, Table 2
	Patient SG11: *‘The top criteria for a transgender person to visit a healthcare professional twice, repeated visits, is not because the doctor can treat their illness; it’s because the doctor uses their ppreferred name. So, it’s not whether the doctor is competent or not; it’s whether the doctor use their preferred name because identity to a transgender person is very important.’*	Q2, Table 2
LGBTQ+ partner affirmation	Patient SG6: *‘I think back to the nurse that dressed my wounds right when she asked me about who that person was, and I came out and told her that she was my partner. And then she asked relevant follow-up questions … So, I felt like she was showing genuine interest in me as a person. And, also, maybe the novelty of being gay. I felt at that point in time, she was really genuinely interested in my, you know, my life.’*	Q3, Table 2
	Student UK8: *‘Being respectful of the situation when you have other people in the room with them, not making assumptions about who that person is to the patient. So, especially with patients who are of certain ages, not assuming it’s their brother or cousin or if they’re a partner and stuff like that. And not assuming that when someone mentions their partner, it’s automatically a heterosexual relationship.’*	Q4, Table 2
Stigma from older generations	Student UK9: *‘Only within the last few years that there has been more awareness of the LGBT community. So, I guess older doctors weren’t taught specifically about LGBT issues or something when they were in training. So, I think it’s important that they try to be more aware about things they should say and do.’*	Q5, Table 2
Understanding of sex vs gender	Patient SG13: *‘I can’t speak for the sexual orientation part, but for gender, I’m not sure to what level they teach medical students about transgender issues or the separation between sex and gender. I think it would be very pertinent to delve a little bit into gender theory, how different people express their gender, the relationship between pronouns and names, and gender expression, presentation, and behaviour. No need to go super in-depth regarding it, but the curriculum should, at the very least, teach the separation between gender and sex.’*	Q6, Table 2

Patients, especially gender-diverse patients, noted that acknowledging patients by their correct names or pronouns was more important to them than the competence of their doctors (Q2, Table 2).

Another area of acknowledging patient identity was partner affirmation (Q3, Table 2). LGBTQ+ patients recounted instances of poor acknowledgement of patient expectations. For example, not acknowledging a partner’s legal rights as next of kin contributed to negative associations of healthcare experiences for LGBTQ+ patients (see Vignette 1). However, only UK students mentioned the importance of LGBTQ+ partner affirmation, stating a need for doctors not to assume heteronormative relationships (Q4, Table 2).

Patients and students both discussed the importance of understanding that gender and sexuality exist on a spectrum. Both groups also highlighted that stigma from older generations of doctors might hinder acknowledgement of patient identity and care (Q5, Table 2). One patient communicated a baseline expectation that medical students should be able to differentiate between sex and gender (Q6, Table 2).

### Health affected by psychosocial factors

Patients felt many psychosocial factors affected their health. Quotations derived from data including sub-themes of having to explain their LGBTQ+ identity to healthcare providers, prejudice in healthcare from wider social factors, lack of consideration from healthcare providers, lack of spousal rights and legal barriers for their LGBTQ+ partners, geographical location, intersectionality, and supportiveness from close ones. There was a mixed appreciation for these factors across the SG and UK student populations. Singapore patients especially expressed a range of concerns about accessing healthcare due to social stigma against the LGBTQ+ community; this included hesitancy to come out to doctors, hindered honesty, and fear of doctors questioning their identity (Q1, Table 3).

**Table 3.** Representative quotes for each sub-theme of the ‘health affected by psychosocial factors’ theme cross-referenced to the corresponding text.

Subtheme	Representative quotes	In-text reference
Having to explain LGBTQ+ makes accessing healthcare more cumbersome	*Patient SG9: ‘Maybe you’re going to doctors once a month. So on a monthly basis, you got to come out to a stranger and tell them your whole history of, like, okay, so I want to be a guy, and this is all the medication I’ve been taking. I’m very sure about this decision. And then what if you encounter a doctor who questions your identity?’*	Q1, Table 3
Prejudice in healthcare arises from wider social factors	*Patient SG13: ‘I think a lot of the issues that are involved in the healthcare system with regard to prejudices are not just exclusively related to healthcare. The root cause doesn’t lie in the healthcare system; it lies more in the general social acceptance of queer issues and the visibility of queer issues.’*	Q2, Table 3
General lack of consideration for LGBTQ+ patients amongst healthcare professionals	*Student SG8: ‘I saw some statistics somewhere: the overwhelming number of doctors in Singapore don’t agree with homosexuality or the LGBTQ+ movement in general*.’	Q3, Table 3
Lack of spousal rights for LGBTQ+ partners and other legal barriers	*Patient SG5: ‘If I am married in a heteronormative relationship, if I go into a coma, my next of kin would be my wife and children, parents, right? But here, he won’t automatically be recognized as my next of kin on papers and legally. So that is a big concern, I think, for many queer individuals in Singapore*.’	Q4, Table 3
Positive experience of affirming UK doctor	*Patient UK7: ‘The resus consultant was finding my partner every half an hour to fill him in, and I don’t know, maybe that happens to everybody, you know, irrespectively, but my partner said he felt really confident and comfortable that he was being reached out to.’*	Q5, Table 3
Geographical location affecting access to specialised sexual health services in UK	*Patient UK13: ‘Just access to sexual health clinics is a lot easier in London, specifically in central London. In Hampshire, access to sexual health clinics – the capacity is really reduced, and it takes longer.’*	Q6, Table 3
	*Student UK10: ‘I think it depends on their location. I would assume that in a lot of parts in London, they meet them very well … But certainly, in rural healthcare environments or in other countries, they would not be as supportive because it is sometimes more discriminatory to people who are transgender specifically. So, there would not be a medium to support them.’*	Q7, Table 3
Intersectionality	*Patient UK13: ‘I don’t think I would have been treated in that way if I was a woman, if I was straight, or if I wasn’t black. Putting those three things or those two things together really changed the type of care that I got.’*	Q8, Table 3
	*Student UK9: ‘I think, especially for POC [people of colour] LGBTQ+, when it’s seen as sort of taboo to be part of the LGBT community, people would struggle more. I guess it depends on the person*.’	Q9, Table 3
Effect of supportiveness of friends and family on mental health	*Patient UK2: ‘I’d been in and out of relationships with men, trying to present myself a certain way for the benefit of certain members of my family who held the belief that I should settle down with a man … It got to the point where I had a suicide attempt because I didn’t want to live in the sort of lie that I was trying to present outwardly when my feelings inside were completely different.’*	Q10, Table 3
	*Patient UK1: ‘I discuss these things with my friends a lot. And it’s great because they’re going through similar processes. And they’re often quite good with advice as well because they’ve gone through the same thing*.’	Q11, Table 3
	*Student UK4: ‘Sometimes, people with LGBTQ may need more help in terms of mental health if, for example, they’re not accepted by the family*.’	Q12, Table 3

Patients were also concerned about societal acceptance and legal recognition of their identity in Singapore affecting their health needs, such as their gender markers. However, they recognised that these might be wider social biases that inadvertently spill into the provision of healthcare (Q2, Table 3). Students generally required prompting to consider psychosocial factors affecting patient health. However, they recognise a general lack of consideration for LGBTQ+ issues among healthcare professionals (Q3, Table 3).

Singapore patients expressed a lack of spousal rights for their partners and more legal barriers affecting access to healthcare for partners on their behalf (Q4, Table 3). Possibly evident of the relative progressiveness of the UK, one patient reflected on a very positive incidence of a doctor affirming their sexuality by repeatedly notifying the patient’s partner of their condition, which made them feel very comfortable with the team (Q5, Table 3).

UK patients were more concerned about other psychosocial factors such as geographical location, where urban places such as London gave patients better access to fertility and sexual health services than rural areas (Q6, Table 3). UK students similarly felt that geographical location could affect healthcare, with sexual and reproductive health being better in London than elsewhere (Q7, Table 3).

UK patients mentioned intersectionality as a concern. Those who were minorities in other aspects of their identity besides their sexual and gender identities (such as race or disability status) felt these intersections compounded and reduced their access to healthcare (Q8, Table 3). Singapore patients also mentioned intersectionality in their societal interactions where ethnic, cultural, racial, and religious identities compounded their LGBTQ+ identities (data not shown). However, they did not explicitly mention intersectionality when describing their healthcare interactions. UK students did recognise that intersectional identities may contribute to greater challenges in meeting the health needs of particular patients (Q9, Table 3).

Patients from both countries described negative experiences of family supportiveness, with transitioning and heteronormative beliefs of their families affecting their mental health (Q10, Table 3). However, friends supported various healthcare needs, from transitioning to sexual health (Q11, Table 3). Students appreciated that lacking family support could negatively affect their health (Q12, Table 3).

### Sexual and reproductive health

This was one domain with significant concordance between patients and students. Patients highlighted the effect of their LGBTQ+ identity on their unique sexual and reproductive health needs (Q1, Table 4), and students demonstrated awareness of this as well, especially for more apparent sexual health needs such as STI (sexually transmitted infections) screening and vaccinations (Q2, Table 4). However, students attributed these needs to factors such as increased sexual activity (Q3, Table 4).

**Table 4.** Representative quotes for each sub-theme of the ‘sexual and reproductive health’ theme cross-referenced to the corresponding text.

Subtheme	Representative quotes	In-text reference
Unique sexual and reproductive health needs	Patient SG7: *‘Because there’s a lot of special treatment that needs to go into treating queer individuals, for example, getting tested for STDs and stuff.’*	Q1, Table 4
	Student UK4: *‘I guess just an increased risk of transmission of sexually transmitted diseases that people of the LGBTQ are more susceptible to.’*	Q2, Table 4
Needs factor from increased sexual activity	Student SG8: ‘*I think the one that always comes up is HIV. And in general, STIs because LGBTQ patients tend to be more sexually active*.’	Q3, Table 4
Sexual health needs well met in UK	Patient UK15: *‘I’ve had some incredibly wonderful healthcare professionals during my time. The support I had during my HIV diagnosis was phenomenal, and the care, support, and attention from the doctors that led that team was incredibly important to how I got through quite bad, upsetting news.’*	Q4, Table 4
	Student UK5: ‘*As far as I know, the sexual health clinics are fairly easy to go to and welcoming. And I know that nowadays, especially with HIV prevention, with both PrEP and PEP, it’s easily accessible.’*	Q5, Table 4
Sexual health provision deficit in Singapore	Patient SG15: ‘*The STI screening has always been in private settings. So, STI screening is actually very, very inaccessible … Then the screenings are really expensive, and insurance doesn’t cover it.’*	Q6, Table 4
Difficulties in accessing fertility services in UK	Patient UK5: ‘*It’s going to be very expensive having children as two women. I know that before the NHS steps in, I’m going to have to spend maybe about 30,000 pounds to try and have a baby myself, and then, if that’s not possible, depending on the area that I live in, that would be dependent on how much IVF rounds I’m allowed, if any. So that’s a big worry.’*	Q7, Table 4
	Student UK6: ‘*There’s obviously clinics set up and all these services in place, say a woman wants to become pregnant in a heterosexual relationship, for example, fertility clinics, and the whole system is set up with that in mind. I guess someone might not be in that relationship; if they want a child, they’re going to face a completely different pathway, and they’re going to need to navigate a different avenue because they want to have kids. I mean, obviously, there’s just a completely different set of needs there.’*	Q8, Table 4

Patients in the UK felt their sexual health needs were well met and recounted supportive and inclusive experiences with sexual health services (Q4, Table 4), and this was echoed by students (Q5, Table 4). Contrastingly, patients in Singapore felt there were significant deficits in the accessibility of sexual healthcare, elaborating that there is limited information on where to access such care and that it is often expensive and not covered by insurance (Q6, Table 4). Students in Singapore did not acknowledge this.

In the UK, lack of sexual health services was not perceived as a concern, but rather the persistent cis-heteronormative assumptions and difficulty accessing fertility services (Q7, Table 4). A few students showed an understanding of these more nuanced needs (Q8, Table 4).

### Mental health

Patients from both countries felt identifying as LGBTQ+ affected their mental health needs, notably anxiety and self-esteem (Q1, Table 5). Hearteningly, students showed insight into the fact that LGBTQ+ patients might have a higher prevalence of mental health issues and further expanded on challenges faced by LGBTQ+ individuals when trying to access support, especially in Singapore (Q2–3, Table 5).

**Table 5.** Representative quotes for each sub-theme of the ‘mental health’ theme cross-referenced to the corresponding text.

Subtheme	Representative quotes	In text reference
LGBTQ+ identity affects mental health needs	Patient SG8: *‘Growing up, constantly thinking that I was doing something wrong on a daily basis, just by being LGBTQ+ or something: that definitely negatively impacted my self-esteem.’*	Q1, Table 5
	Student SG8: *‘I know for a fact that depression and anxiety are much higher among LGBTQ patients.’*	Q2, Table 5
	Student SG10: *‘There aren’t really a lot of avenues within Singapore for people to seek help unless, I guess, they go to private health clinics or, especially for psychological health, because mental health is something that even for a lot of cisgender or straight people, they also have troubles with looking for avenues to address their own mental health needs. So, I think for LGBTQ+ people, it’s even worse because of all these constitutions and how it doesn’t serve the LGBTQ+ people.’*	Q3, Table 5
Trans identity and mental health needs	Patient SG11: *‘Because the time I take a lot of hormones, I think it’s the side effect of the hormones. So I wear pyjamas every day for half a year. I never go out; I even go to see night shows in my pyjamas. That’s the only mental thing.’*	Q4, Table 5
	Student UK6: *‘Maybe also much higher rates of mental health issues in these [transgender] patients, and a lot of this might be tied up to experiences that they’ve had in life, whether it’s negative or positive. You know, a lot of the time, it’s negative. That leads to higher rates of depression, anxiety and even suicidality in these patients.’*	Q5, Table 5
Insufficient mental health training	Patient SG2: *‘It seems that very few of them are actually trained in queer healthcare. Some of them: they don’t know what to do, or they don’t respect queer people properly.’*	Q6, Table 5
	Patient SG7: *‘I could talk about anxiety … and suddenly, they would relate it to my queerness out of nowhere. And obviously, that talk is more about their perception of queerness than it is about my identity as being queer.’*	Q7, Table 5
	Student UK6: *‘You have these interventions for patients, but I think unless you have a psychiatrist or psychologist who specialises in treating people with gender identity issues, then that might not be as effective for them.’*	Q8, Table 5
Allyship from mental healthcare professionals	Patient SG10: *‘I just felt more comfortable sharing with my therapist because I knew that, oh, my therapist was recommended to me by a queer friend. So I kind of knew they’re gonna be queer-friendly.’*	Q9, Table 5
	Student SG6: *‘Things like specific psychiatric services that are very understanding and non-stigmatising towards LGBT identity.’*	Q10, Table 5

Gender-diverse patients discussed that hormone replacement therapy could affect their mental health and trans identity (Q4, Table 5). Likewise, students identified the gender-diverse population as requiring more mental health support, with long waiting times and doctors inadequately trained to help them before reaching specialist services (Q5, Table 5).

Lack of training to address mental health needs was an issue in Singapore (Q6, Table 5). Further, doctors would often extrapolate mental health issues to patients’ LGBTQ+ identities (Q7, Table 5). Students demonstrated awareness of this gap and felt that traditional mental health interventions might not be as effective unless healthcare professionals were explicitly trained in LGBTQ+ mental healthcare and developed more nuanced understandings of the community (Q8, Table 5).

Positive experiences, including allyship with mental health healthcare professionals, made patients feel comfortable coming out (Q9, Table 5). Students also noted positivity in service provision within specific psychiatric services (Q10, Table 5).

### Gender-diverse health

Gender-diverse patients had negative and uncomfortable experiences within the healthcare system and its providers. Patients described discrimination and transphobia from doctors, who implied gender identity was a mental health issue (Q1, Table 6). They also shared instances where doctors lacked technical knowledge about caring for gender-diverse patients (Q2, Table 6). They communicated that doctors often did not address or interact with them appropriately (Q3, Table 6).

**Table 6.** Representative quotes for each subtheme of the ‘gender-diverse health’ theme cross-referenced to the corresponding text.

Subtheme	Representative quotes	In text reference
Gender identity as a mental health issue due to discrimination and transphobia	Patient SG1*: ‘The concern I have is when I inevitably have to tell that I am transgender, or if I’m visibly transgender, maybe a year or two from now, then something happens to me. My biggest concern would be how they will end up treating me after I tell them I am transgender. There was somebody who went for their initial consultation, and the psychiatrist with them was more or less everything I would fear. For the initial appointment, the doctor was transphobic, so he would give statements which were very dismissive of the gender identity portion and really focused on the entire issue as a mental health issue to be solved.’*	Q1, Table 6
Lack of knowledge in caring for trans patients	Patient SG2: *‘Some diseases affect males and females differently. And sometimes it depends on which part of you is “male” and which part of you is “female”, right? Some of them is controlled by hormones and stuff. So, they will be very confused on what to do to you, especially if you’re trans on HRT and stuff. But you can tell the doctor didn’t know. There was a very clear level of confusion with the doctor, even though she is the senior doctor in that department.’*	Q2, Table 6
	Patient SG2: ‘*I need to go for X-rays and don’t know which changing room to put me in, and they didn’t tell me to take off my bra for the X-ray. They didn’t tell me that for some reason. They don’t want to use their eyes; they just want to look at the paper … But I saw when they talked to other female patients that they did tell them to take off their bras*.’	Q3, Table 6
	Student SG7: *‘I don’t know much because, honestly, I have not known any transgender people in my life.’*	Q4, Table 6
Trans healthcare barriers within health infrastructure	Patient SG11: *‘How about the infrastructure? Is there a non-binary toilet? Don’t ask me to use the disabled toilet; I cannot take it … Because I’m not disabled; I’m more able than you. I’m more empowered than you.’*	Q5, Table 6
	Patient UK15: *‘I think we still have very binary services where, if you think about cervical cancer screening, the system should include trans men and the ones who’ve got a higher rate of cancer and transmission … If services are not set up to be inclusive, for things like cervical cancer screening, if you exclude [trans people], it can be to the detriment of them.’*	Q6, Table 6
	*Patient SG1: The only thing that I want to see is more doctors and psychiatrists who form part of the gender care clinic; right now, it’s run by one psychiatrist – and because of this, the waitlist is seven months long. If the waiting time maintains between now and December, and it doesn’t increase, my hormone therapy will only start sometime in July next year.*	Q7, Table 6
	Patient UK1: *‘What I’d really like to see is specifically the trans healthcare moving to an informed consent model. My friend who transitioned in the 1970s could do it with a single meeting with her GP. My friends in America can do it with a single meeting with their GP. For my friends, who transitioned in 2009 when I was at university, it was six months from talking to a GP to getting to the gender clinic. And now it’s an average of six years, if not more.’*	Q8, Table 6
	Patient UK15*: ‘I think there’s a shortage of centres; whether we have these very specialised clinics where there are massive waiting lists, or should we actually have more accessible services in the local area?’*	Q9, Table 6
Costly and inaccessible gender-affirming surgeries and HRT in public healthcare	Patient SG7: *‘Operations for trans individuals is not something that’s talked about in Singapore because most trans individuals would get it done overseas because [in Singapore] either you need to go to someone’s private practice, which costs 15 to 20k easily.’*	Q10, Table 6
	Patient UK11: *‘I’ve heard some really difficult experiences shared by my trans friends. I think healthcare just seems to be really horrendous for trans people trying to access healthcare. For example, I’ve had friends not being able to get access to top surgery and having to fundraise.’*	Q11, Table 6
	Student SG7: *‘Sexual reassignment surgery is also not allowed in Singapore. I mean, not not allowed, but you can’t do it in Singapore, but you can do it in other countries.’*	Q12, Table 6
	Student UK8: *‘Instead of having to order DIY [do it yourself] hormones off the internet and stuff like that. So that’s quite rampant right now where people are having to order their oestrogen and testosterone and figure out a dosage that agrees with them.’*	Q13, Table 6
Exclusion from public health initiatives	Patient UK1: *‘The NHS is so, so bad … I’m really worried about not being invited to a cancer screening or someone not noticing it, or there being a problem with the hormones I’m taking, which I don’t realise in time, and no one cares enough to follow up about it. So, I guess I come under medical neglect. I’m worried about dying as a result of some bizarre medical neglect brought on by being trans.’*	Q14, Table 6
Need for training to improve communication with gender-diverse patients	Patient SG11: *‘Train! Besides training the doctors, train the front desk, please – the receptionist, please. Train them why it’s so important to use pronouns; train them why it’s so important to use preferred names. Train your front desk to have a rapport with us, to make us feel comfortable to visit them again. If not, we all will just choose a coping mechanism. Last time, the coping mechanism was drugs. Or just self-medicate like last time. So, really hope that besides training your healthcare professionals, don’t forget the front desk.’*	Q15, Table 6
	*Student SG7: ‘I guess when people are transitioning, it may also be a very confusing point in their lives. So maybe we could reach out to them more as healthcare professionals.’*	Q16, Table 6

Certain students’ responses mirrored the gap in understanding seen in doctors’ interactions with our patients. Most students did not acknowledge gender-diverse health until prompted, and students who required prompting had a worse understanding of the themes discussed overall. Also, there was uncertainty around how well gender-diverse health needs were being met, with some having no clinical exposure to gender-diverse patients (Q4, Table 6).

Regarding infrastructure (Q5, Table 6), patients cited exclusionary services (Q6, Table 6), lengthy wait times (Q7, Table 6), convoluted referral pathways (Q8, Table 6) and shortages of gender care clinics (Q7 & Q9, Table 6) as substantial barriers to accessing gender-diverse healthcare in both countries. These barriers were coupled with the costly and inaccessibility of gender-affirming surgeries and HRT in Singapore (Q10, Table 6) and the UK (Q11, Table 6) via public healthcare systems.

Encouragingly, certain students acknowledged HRT and gender reassignment surgery as topics in gender-diverse healthcare and demonstrated an understanding of barriers to accessing such care (Q12, Table 6). Students were also aware of the hormonal side effects of HRT and the need for patients to self-prescribe HRT due to its unavailability in the public health systems (Q13, Table 6). However, patients voiced concerns about not being accounted for in public health initiatives such as cancer screenings (Q6 & Q14, Table 6). Further, patients shared hopes for better communication with gender-diverse patients, suggesting training for support staff and healthcare professionals to refer to patients correctly (Q15, Table 6).

Overall, there were discrepancies in students’ perceptions of the accessibility of gender care and the sufficiency of such care. Certain students thought more services and specialists were needed and recognised the impact on patients waiting to receive gender reassignment surgery and other specialist gender care (Q16, Table 6). In contrast, others felt they were already well accommodated in the current system.

### Support and professionalism

A vital contributor to a patient’s experience and opinions of the healthcare system is the workforce providing that care. Many patients described the need for doctors to create non-judgemental environments since they may be hesitant to share necessary information related to their health needs due to their perception (Q1, Table 7). Students felt it was imperative to take a non-judgemental approach, including recognition of their own biases, especially when patients share sensitive information such as their sexual identity and personal lives. They felt it was important that personal beliefs do not hinder patient care (Q2, Table 7). Furthermore, students echoed concerns about the expression of personal opinions, inferring that discrimination, assumptions and stereotypes could deter LGBTQ+ patients from disclosure (Q3, Table 7).

**Table 7.** Representative quotes for each sub-theme of the ‘support and professionalism’ theme cross-referenced to the corresponding text.

Subtheme	Representative quotes	In-text reference
Non-judgemental environment, avoiding visible displays of religiosity or discrimination	Patient SG5: ‘*Your comfort level with my queerness as a medical professional would determine how much I tell you. And if I had any slight semblance or judgment that you might be judgmental, or you might not understand, then I might not be truthful with telling you my sexual health history, and that has to change.’*	Q1, Table 7
	Student SG5: *‘You must also make sure that you take a non-judgmental approach to patients. This is the same for all patients also, but maybe more so because people are sharing a bit about their sexual identity and their personal life with you, then you must separate your own religious beliefs from how you want to treat your patients.’*	Q2, Table 7
	Student SG2: *‘I noticed that even within my batch, when it gets to discussing or referring to LGBTQ+ people, there is an air of disapproval, and I guess everyone can have their own personal opinion, but when it comes to patient-doctor relationships, this mix-up or this thought taints that relationship then it will be quite difficult to give proper health care.’*	Q3, Table 7
Avoiding cis-heteronormative assumptions	Patient UK14*: ‘I think that doctors need to be open to the fact that someone may not be the typical presenting gender or sexual identity and understanding of those particular needs. I guess, as I mentioned, not assuming that everyone coming in is going to be cis[gender] and straight.’*	Q4, Table 7
Pre-conceived notions from older generations	Patient SG11: *‘It’s the older generation, the World War II descendants: a bit set in their way … I’m waiting for them to die first or retire. And then, you know what? They don’t retire; the older generation never dies.’*	Q5, Table 7
	Student UK4: *‘There are more traditional older generation doctors; they [the patients] felt like they [the doctors] are not accepting them as much, particularly in areas outside of London. So, learning that perspective and realising that perhaps in the healthcare system, we are not as inclusive of the LGBTQ community as we could be.’*	Q6, Table 7
LGBTQ+ affirming doctors	Patient SG8: *‘I asked her directly what her views on the LGBTQ+ community are, and she’s like: Oh, I’m very accepting. I have clients who are LGBTQ+ as well … assurance that your doctor is not going to give you a compromised sort of experience seeking healthcare, even though you’re not straight, is definitely an experience that I think would make a lot of queer people a lot more comfortable seeking help.’*	Q7, Table 7
Visible identification of LGBTQ+ friendly doctors	Patient UK15: *‘If you walk into a place and see a rainbow lanyard and someone with their pronouns badge on, there is a mission statement about values and ethos, and it’s reassuring. I think people think it’s just paying lip service and think it’s tokenistic in its approach. It is actually a very good statement of the care people intend to provide. It’s incredibly welcoming.’*	Q8, Table 7

Patients and students alike highlighted the need for doctors to minimise preconceived perceptions, especially about gender and sexual identities, and avoid the assumptions of cis-heteronormative relationships (Q4, Table 7). Further, patients had strong opinions regarding preconceived notions about LGBTQ+ patients from older generations of doctors (Q5, Table 7). Equally, students appreciated the impact of older-generation doctors’ non-acceptance of LGBTQ+ identities in their clinical practice (Q6, Table 7).

Patients’ experiences of positive interactions with LGBTQ+ affirming doctors involved instances where their doctors could provide assurance their healthcare would not be compromised based on their identity (Q7, Table 7). Patients from both countries and students felt that visible identification of LGBTQ±friendly or LGBTQ±identifying doctors in online and face-to-face settings, such as rainbow lanyards and pronoun badges, was necessary and a positive mechanism to affirm LGBTQ+ patients (Q8, Table 7).

### Medical school teaching

Students were asked about the teaching they received and its adequacy. UK students described some aspects of current education (Q1, Table 8), but much knowledge was gained outside formal teaching (Q2, Table 8). In their early years, Singapore students had general clinical communication sessions that briefly mentioned topics such as being non-judgemental and open-minded and when to ask appropriately about gender and sexual identity (Q3, Table 8). However, most students believed their teaching on LGBTQ+ healthcare was inadequate, especially in Singapore (Q3, Table 8).

**Table 8.** Representative quotes for each sub-theme of the ‘medical school teaching’ theme cross-referenced to the corresponding text.

Subtheme	Representative quotes	In-text reference
Some teaching but much knowledge from outside of formal teaching	Student UK2: *‘The only thing medical school taught was one lecture in our endocrinology section, just about what hormones transitioning people take. But that’s about it.’*	Q1, Table 8
	Student UK9: *‘It’s mostly been from my own research. As far as I’ve seen, I don’t think they teach it enough.’*	Q2, Table 8
	Student SG7: *‘There’s actually one session … about not assuming things and keeping an open mind. And she actually brought in the issue of LGBT and also how to address trans patients and all that, which I think was a really good move. But it was really very short because she didn’t have a lot of time.’*	Q3, Table 8
Cis-heteronormative assumptions	Student UK7: *‘Basically, we’re assuming that every single patient that walks through our doors is straight/cis. There’s never really anything that the medical school tests you on.’*	Q4, Table 8
Dismissing gender pronouns in favour of biological sex	Student SG4: *‘Because they don’t teach it at medical school, at least not that I’ve seen so far. In one of our tutorials, somebody brought up the “he/she” pronoun thing, and then the doctor said, oh, for the sake of simplicity, we just go by biological gender; otherwise, you open a whole can of worms.’*	Q5, Table 8
Want more clinical communication sessions	Student SG8: *‘But we have no clinical communication lessons with specific groups of patients … like disenfranchised and marginalised groups, which include the LGBTQ community, which is really important because the health issues may change, and the topics of discussion may change as well … And I think it’s important to have training on talking about issues related to the LGBT community, because regardless of if you believe that being homosexual or transgender is right or wrong, they are at the end of the day patients, right?’*	Q6, Table 8
Did not feel adequately trained	Student UK5: *‘Never had any teaching on it. I’ll be honest: if I had suddenly, during a GP [general practice] placement, had an LGBTQ+ patient come in or someone who’s non-cisgender, I’d be fairly lost and wouldn’t know how to support them.’*	Q7, Table 8
Societal and policy changes also needed	Student SG6: *‘I think sometimes the issues stem very deep in the way society’s structured, which is very unfortunate because sometimes members of the public might just see HIV as a disease that can only affect gay men when that’s actually not the case. I think on a very large scale, it’s gonna take quite a lot of effort: it’s not just from medical professionals, it’s also from schools educating, or even policy changes.’*	Q8, Table 8

UK students highlighted the implicit assumption in medical school that all patients were cishet and that students lacked the means to interact with a non-cisgender patient (Q4, Table 8). Additionally, students from both countries cited negative experiences, such as doctors at placements dismissing the use of gender pronouns in favour of biological sex (Q5, Table 8).

Students felt that teaching about LGBTQ+ health could be improved and wanted clinical communication sessions with LGBTQ+ individuals (Q6, Table 8). They explicitly stated that medical schools were responsible for teaching LGBTQ+ health as doctors-in-training will inevitably interact with LGBTQ+ patients in their medical careers and that they did not feel adequately trained to treat LGBTQ+ patients (Q7, Table 8). However, some students acknowledged that societal and policy changes were also needed (Q8, Table 8).

## Discussion

LGBTQ+ patients’ narratives of their healthcare experiences showed most were dissatisfied with healthcare related to their gender and sexual identities, and many described discrimination from doctors. Patients’ experiences in Singapore compared to the UK largely aligned with the more conservative societal norms and attitudes towards LGBTQ+ individuals, but students were more affirming towards their experiences and perspectives, perhaps reflective of the improving societal attitudes in both countries towards LGBTQ+ individuals. As future change-makers, medical students are well-placed to develop identity-affirming behaviours, but many lack a comprehensive understanding of LGBTQ+ health experiences and needs. Our study demonstrates the need to improve both the healthcare environment for LGBTQ+ individuals and the undergraduate medical education to foster sustained change.

Students lack an understanding of the complex sexual and reproductive health needs identified by patients in this study. Although many students volunteered responses regarding sexual health, their knowledge was limited to superficial views of HIV and PrEP [30], suggesting medical training may be unconsciously influenced by common LGBTQ+ stereotypes rather than a deep understanding of health needs. Perpetuated stigma was further noted from Singapore students’ views that STIs in the LGBTQ+ community were associated with increased sexual activity and more traumatic modes of sex. Knowledge gaps were also found through students’ unawareness of the poor availability of sexual health resources in Singapore, an issue repeatedly reported by patients, highlighting the need for students’ biases and knowledge to be addressed prior to interacting with LGBTQ+ patients. This is in stark contrast to the UK, where both patients and students described adequate sexual health resources, and issues were more focussed on the delivery of that care. Other problems, such as fertility and gender-diverse healthcare, were prominent for patients in both our study countries but seldom mentioned by students, underlining the need for enhanced training for future doctors on LGBTQ+ patients’ sexual health issues, as identified by non-governmental organisations in Singapore [18]. Our study identifies limited coverage of gender-diverse health needs at medical school, adding nuanced narratives to our previous findings from a cross-sectional survey in Singapore and the UK [19]. The poor clinical experiences of gender-diverse patients can be corrected by improved education and medical training to provide affirmative care for this heavily marginalised group [31].

Some patient experiences, such as geographical disparities in healthcare access, were known to students, especially in the UK. Such disparities may explain inequalities in life expectancy across geographical areas [32]. Patients who identified with other marginalised groups outside their LGBTQ+ identity expressed compounded difficulties when accessing healthcare, corroborating a previous Singaporean study showing that intersectionality worsened patients’ mental health [33]. These findings echo a need identified in nursing education for greater programmatic inclusion of LGBTQ+ content with parallel topics such as social determinants of health and intersectionality [34]. Patients also described significant but poorly met mental health needs, which students identified. This alignment of perceptions may suggest interactions of students with LGBTQ+ patients, perhaps during clinical placements, where they notice such disparities firsthand [35], but may also arise from time spent on social media [36].

Our study suggests that unconscious pre-existing cis-heteronormative assumptions, rather than deliberate discrimination, inherently affect and damage the care that LGBTQ+ patients receive, often to a concerning extent. It has been shown that failures by doctors to discuss sexual orientation with patients openly hinder patient education and affect patients’ health outcomes [37]. In our study, misgendering and a lack of recognition of sexual and gender diversity were common patient experiences and were perceived as judgemental, unsupportive and unprofessional. Patients and students expressed concern towards ‘older generations’ of doctors, who were perceived to have less tolerant views. This specific expression used by interviewees may be considered ageism, but it is important to acknowledge the differences in the prevailing culture, paradigms, and training experienced by previous generations since these may inform doctors’ contemporary practice. Differences in attitudes among younger doctors are likely due to increased tolerance for LGBTQ+ individuals in younger societies, as found in Southeast Asia [38]. This generational gap also forms part of the argument for younger healthcare educators to train medical students on LGBTQ+ healthcare, being better placed on what is needed to improve the healthcare environment for LGBTQ+ individuals. All interviewed students in both countries expressed positive attitudes toward learning about and supporting LGBTQ+ patients’ health needs, despite many students lacking specific knowledge or expressing perceptions which were misaligned with patients’ reported experiences. This suggests that students are well-placed to be the future changemakers to improve equity in LGBTQ+ healthcare, particularly in Singapore.

Medical school remains a crucial context where health educators can create meaningful opportunities to instil thoughtfulness and address ignorance in future doctors, challenge cis-heteronormative assumptions, and lead to positive behaviours for our patients. Previous studies showed minimal coverage of LGBTQ+ health in medical school teaching in the UK and Singapore [19,39,40], and students in our study echoed this. Importantly, students can drive change in the education system in concert with patients [41]. Whilst medical educators can introduce third-sector or community-based groups to tailor interventions to the needs of local LGBTQ+ populations [42], bringing patients and students together can stimulate the creation of tailored training programmes for LGBTQ+ care [23]. In socially conservative societies, even a single session of sensitivity training, as students in this study suggest, can broaden perceptions [43] and enhance students’ confidence in communicating with gender-diverse patients [44]. Simulated sessions with LGBTQ+ patients [45] may promote culture change through the collective action of patients and students. Such opportunities may humanise patients from communities where students may not have had personal encounters [46] and help them develop greater empathy for this group of patients.

There is also a need to go beyond curriculum reform to improve LGBTQ+ health provision, corroborating findings from a previous Singaporean study investigating LGBTQ+ non-governmental organisations and clinical medical student perspectives [40]. Moreover, there is evidence from the UK, Singapore and elsewhere to suggest that structural stigma can worsen the health and well-being of LGBTQ+ individuals [47]. Training alone cannot significantly impact behavioural changes towards minoritised groups without system-level improvements regarding diversity, equality, and inclusion [48]. Since medical students spend considerable time in the clinical workplace [49], formulating understandings of sociocultural norms [50], professional role modelling has a major influence on student development [51]. Healthcare professionals and placement providers can play a role in medical students’ hidden curriculum and understanding of structural stigma by role-modelling an equitable and unbiased workplace culture. In Singapore, calls to strengthen certain aspects of social work provision for LGBTQ+ individuals [52] may also be applied to medical doctors. Training for doctors to improve behaviours towards LGBTQ+ patients could create a welcoming and inclusive clinical environment for students to model based on the theory of planned behaviour [53]and can contribute to medical students’ understanding of LGBTQ+ health needs by integrating didactic medical school teaching with clinical experiences, which is more effective than having one-off sessions in the curriculum [45]. Additionally, although patients described affirming strides in the visibility of LGBTQ+ inclusiveness, such as rainbow lanyards and pronouns badges, they wished for gender-neutral language in forms and communication to better acknowledge their identities [54]. Training and identifying LGBTQ+ health-competent or ‘friendly’ doctors [55] may provide educational role models for medical students and further improve patient comfort, as patients in our study suggested. These ideas corroborate the inclusion framework suggested by the Health and Care LGBTQ+ Leaders Network in the UK [56] and further suggestions to improve health provisions from the British Medical Association [57].

The contrast between Singapore’s socio-political landscape for LGBTQ+ individuals and the UK’s more socially progressive landscape plays a large part in how well-informed medical students are and affects the experiences and subsequent health outcomes of LGBTQ+ patients [58]. Whilst societal acceptance is improving in countries like the UK, with movements to enhance health equity for LGBTQ+ patients [59], sociolegal recognition of LGBTQ+ individuals is still lacking in countries like Singapore [60]. Our findings echo previous calls for the better protection of LGBTQ+ rights in Singapore to enable equal healthcare treatment, such as changes to laws for gender markers [40].

A limitation of this study is the potential selection bias of interviewees, where those with prior interests in LGBTQ+ healthcare or who had specific positive or negative experiences to share may have been more inclined to participate. Thus, our populations’ perceptions and understanding may be skewed compared to the baseline student and LGBTQ+ community populations. Additionally, there may have been a gender bias in our interviewees recruited from a consecutive sampling approach, where gender-diverse patients may have been under-represented in the UK compared to Singapore. However, both groups reached meaning saturation through thematic analysis, suggesting that the study populations were homogeneous. The absence of other selection bias factors indicates that our populations were sufficiently large to represent the general LGBTQ+ and student communities. Further bias may have been introduced by investigators when coding the student interviews with knowledge of previous themes from the patient interviews, and quotes may have been unintentionally coded to specific themes. However, this allowed for uniformity and enabled easier comparisons of the themes between populations. Finally, as with any qualitative research, interpretations of interviews and results were subjective but were minimised with five co-investigators from both countries simultaneously analysing data and formulating this article, thus bringing different perspectives to minimise bias.

In conclusion, LGBTQ+ patients described numerous negative experiences in healthcare, particularly about cisgender-heteronormative assumptions from their doctors, as well as healthcare services which do not affirm, or even discriminate against, their gender and sexual identities. In many respects, patients’ experiences and needs were seen by medical students, even if at a superficial level. In areas with deficits between patients’ and students’ perspectives, students demonstrated a willingness to learn and improve the healthcare environment for LGBTQ+ individuals. Areas for improvement in medical education encompass inclusive training from curricular content and professional development, incorporating both didactic teaching and simulated learning to improve LGBTQ+ health insights. Actionable outcomes can be achieved by bringing patients and students together to reach culture change, which would otherwise be difficult on an individual level. Changes at an infrastructural level would complement and advance the training in medical education, and unfavourable assumptions and attitudes toward LGBTQ+ individuals must be addressed at the societal level to improve healthcare experiences for this marginalised population.

## Supplementary Material

Supplemental Matieral 1.docx

Supplemental Material 2.docx

## Data Availability

This manuscript used only original data. The authors do not have ethical approval to share the original interview transcripts. However, where detail can be made available without the risk of breaching individuals’ anonymity, for example, by revealing highly specific anecdotes, the authors are willing to make certain parts of the interview transcripts available on request.
