# Supplementary material for: Patient voices and student insights into LGBTQ+ healthcare: a call for equitable healthcare through medical education
Source: Med Educ Online. 2024 Sep 17;29(1):2405484. doi: 10.1080/10872981.2024.2405484 (PMC11409410; doi:10.1080/10872981.2024.2405484)
Supplement: Supplemental Matieral 1.docx [file ZMEO_A_2405484_SM1693.docx]

**LGBTQ+ Patient Interview Guide**

*Interviewer – check consent form has been received*

| # | Main question | Side questions |
| --- | --- | --- |
| Just to find out briefly about your experiences as LGBTQ+... | | |
| 1 | **Could you tell us a bit more about yourself?** | — anything that you’re comfortable sharing with us.  [If prompt needed: For example, your work, education, passions/hobbies, or family?]  If not given,   - Age - Occupation - Gender identity |
| 2 | **What part of the LGBTQ+ community do you identify as? (if not specified)** | [Could you describe it in more detail? IF unfamiliar/ unconventional identity]  *Do you also identify as any other gender or sexual minority (diverse?) group?*  How long have you considered yourself as such?  Have you ever had a different [gender/sexual] identity from now, or questioned your identity?  Are there other parts of your identity that have any impact on your experiences as an [LGBTQ+] person?  *[e.g. if prompt needed: ethnicity/religion/culture/language/age/occupation]* |
| 3 | **Do you feel that identifying as *[insert specific LGBTQ+ group]* affects your day-to-day life?** | → prompt with specific areas of daily life, e.g. at home, at work, out in public  Are you out to anyone?  Who are you out to?  Are they supportive?   - family - friends/coworkers   [If support or LGBTQ+ peers not mentioned] *Do you know others who share a similar sexual/gender identity as you?*  Are there any spaces where you feel more/less comfortable/safe as an [LGBTQ+] person?  [If not supportive]  *What challenges do you face because of this lack of support? (paraphrase as needed)* |
| Segue: talking about health   - **What are some of your health needs in general, at the moment?**    - Are you seeking medical treatment for anything? | | |
| 4 | **Do you feel that identifying as [LGBTQ+] affects your health and health needs?**  **[If no mention of mental health, ask about mental health, and other prompts]** | If so, how?  What unique healthcare needs do you think you have being LGBTQ+?  [**Prompts**]  Sexual health: STI screening/checkup, prevention advice, sexual dysfunction, anal injury  Mental health: dealing with stigmatisation, acceptance of orientation and identity, availability of correct educational and support resources  Sex assignment: indications, procedure  Blood donation? |
| 5 | **Would you feel comfortable sharing these health concerns with your doctors?** | Do you think your [above] health needs are being met?  Ask if they can provide examples if possible  Any particular settings where the care you receive for these health issues is better, or worse?  How often do you disclose your sexual/gender identity to your doctors?  Would you feel comfortable sharing these healthcare concerns with your family/loved ones/partner?  In an ideal situation, what role do you wish your caregivers could play in your healthcare? |
| 6 | **Is there anything that worries or bothers you when you interact with healthcare providers?**  [If not mentioned] How about for doctors? | Confidentiality wise - are you worried?  Have there been any moments where you felt discriminated against or didn’t feel comfortable sharing with a doctor?  What might hold you back from being open about your sexual orientation/gender identity with healthcare providers? How about from sharing about the issues that you face as a result of your identity?  **What do you think the healthcare provider could have done differently for you?** |
| 7 | **Conversely, have you had any positive or affirming experiences when seeking healthcare?**  [If not mentioned] How about for doctors? | Have there been any moments where a doctor contributed to this positive experience?  All in all, have you noticed any change in your healthcare experiences over the past (10?) years?  e.g. access to better healthcare services, changes in attitudes of healthcare professionals, systemic changes |
| 8 | **Have you heard of any stories or opinions about healthcare from someone else who is LGBTQ+?** | e.g. friends, partners, family members  If in healthcare — what have your experiences been like with patients who are LGBTQ+? |
| 9 | **How would you hope for healthcare to be different in the future?** | [Make reference to experiences that they may have shared earlier]  Is there anything you would like to say to our fellow medical students? Do you have any advice for them? |

**After interview**

Thank them for their time, offer them further updates about this project, signpost support if needed
