# Supplementary material for: Patient voices and student insights into LGBTQ+ healthcare: a call for equitable healthcare through medical education
Source: Med Educ Online. 2024 Sep 17;29(1):2405484. doi: 10.1080/10872981.2024.2405484 (PMC11409410; doi:10.1080/10872981.2024.2405484)
Supplement: Supplemental Material 2.docx [file ZMEO_A_2405484_SM1692.docx]

**LGBTQ+ Student Interview Guide**

*Interviewer – check consent form has been received*

| # | Main question | Side questions |
| --- | --- | --- |
| 1 | **Could you tell us a bit more about yourself?** | What are your pronouns/gender?  What year have you completed in medical school?  Do you identify as part of the LGBTQ+ community? |
| 2 | **What specific health needs do you think LGBTQ+ people have?**   - Health needs relating solely to sexuality/gender   Vs.   - Health needs not related to being LGBTQ+ but is prevalent in the community | Can you tell us more about your perceptions of this health need?  How well do you think these needs are being met by healthcare providers? |
| 3 | **What specific aspects of the patient consultation should a doctor be aware of when speaking to an LGBTQ+ identifying patient?**  *(e.g. don’t misgender, ask for pronouns, don’t assume the gender of partner/caregiver)* | Is there anything doctors should ask or keep in mind of during the consultation? |
| 4 | **What do you understand about ___ as a health need?**  [If no mention of non-cisgender health, acknowledging patient identity, mental health, support and professionalism (how do you think doctors can support LGBTQ+ patients in terms of professionalism?), ask about the above] | Can you elaborate on your understanding of sexual and reproductive health and how it may affect LGBTQ+ patients?  How well do you think these needs are being met? |
| 5 | **Do you feel the teaching you received at medical school adequately prepares you to address the aforementioned health needs?**  [based on Q3, Q4] | Ask if not past 15 minutes  health needs being: non-cisgender health, acknowledging patient identity, mental health, support and professionalism.  Do you think the medical school should address these needs or not?  If yes and not mentioned already→ What could the medical school do about it? |
